# Enhancing Urban Data Analysis: Leveraging Graph-Based Convolutional Neural Networks for a Visual Semantic Decision Support System

**DOI:** 10.3390/s24041335

**Published:** 2024-02-19

**Authors:** Nikolaos Sideris, Georgios Bardis, Athanasios Voulodimos, Georgios Miaoulis, Djamchid Ghazanfarpour

**Affiliations:** 1 Department of Informatics and Computer Engineering, University of West Attica, 12243 Athens, Greece; nsideris@uniwa.gr (N.S.); gmiaoul@uniwa.gr (G.M.); 2 School of Electrical and Computer Ingineering, National Technical University of Athens, 15780 Athens, Greece; thanosv@mail.ntua.gr; 3 Faculty of Science and Technology, University of Limoges, CEDEX, 87060 Limoges, France; djamchid.ghazanfarpour@unilim.fr

**Keywords:** convolutional neural networks, urban planning, machine learning, decision support, graph visualization

## Abstract

The persistent increase in the magnitude of urban data, combined with the broad range of sensors from which it derives in modern urban environments, poses issues including data integration, visualization, and optimal utilization. The successful selection of suitable locations for predetermined commercial activities and public utility services or the reuse of existing infrastructure arise as urban planning challenges to be addressed with the aid of the aforementioned data. In our previous work, we have integrated a multitude of publicly available real-world urban data in a visual semantic decision support environment, encompassing map-based data visualization with a visual query interface, while employing and comparing several classifiers for the selection of appropriate locations for establishing parking facilities. In the current work, we challenge the best representative of the previous approach, i.e., random forests, with convolutional neural networks (CNNs) in combination with a graph-based representation of the urban input data, relying on the same dataset to ensure comparability of the results. This approach has been inspired by the inherent visual nature of urban data and the increased capability of CNNs to classify image-based data. The experimental results reveal an improvement in several performance indices, implying a promising potential for this specific combination in decision support for urban planning problems.

## 1. Introduction

### 1.1. The Crucial Impact of Urban Planning on City Development

Urbanization has been rapidly increasing in recent decades [1], and, as noted by the United Nations, this is predicted to increase further in the forthcoming years, especially in developed countries where the proportion of the urban population in relation to the rural population is overwhelmingly high. It is an expected outcome to also witness a notable surge in both the importance and the intricacy of urban planning [2,3]. Urban environments are dynamic and multifaceted, and they involve complex interactions among components like buildings, infrastructure, transportation systems, and human activities.

The unceasing expansion of cities to accommodate burgeoning populations presents an increasingly formidable challenge to a diverse array of stakeholders and professionals, including civil engineers, sociologists, environmental experts, and municipal authorities [4]. This interdisciplinary process demands intricate interconnections among its various subsystems, spanning a wide array of domains such as legal frameworks, socio-political dynamics, financial investments, and also computational analyses. A primary challenge inherent to urban planning and development lies in its inherently constrained margin for error and iteration. The advent of urban big data has led to an unprecedented inflow of heterogeneous and voluminous data from various sensors.

### 1.2. Urban Planning Challenges

The challenges associated with this data influx are numerous. Urban data sources such as satellite imagery, IoT devices, and administrative records produce large amounts of information that differ in format, scale, and quality. These diverse data are difficult to integrate and consolidate, making it hard to pertain a holistic view of the urban landscape. The visualization and interpretation of such data thus become complex due to their high dimensionality requiring advanced data analysis techniques [5]. Additionally, feature selection challenges, dimensionality reduction problems, and the need for sophisticated predictive models hinder optimal utilization of urban data [6]. This section explores the multifaceted challenges in urban data analysis, emphasizing the need for innovative approaches that can handle the unique characteristics of urban data.

The disparate character of the data sources makes urban data analysis even more complicated. These data sources usually have an increased level of spatial and temporal granularity, while the different urban data types exhibit certain distributional characteristics. Consequently, various kinds of data require a robust framework with the ability to handle different data formats, scales, and structures, especially for multi-modal, multi-scale, and multi-temporal data representations [5]. Besides this fact, Internet of Things (IoT) devices and remote sensing technology have resulted in an increased volume and velocity of urban data [7]. The latter poses, in turn, issues related to the quality, accuracy, and consistency of the data, which call for one or more preprocessing stages aimed to ameliorate them. In general, the aforementioned issues may be addressed through feature selection and relevant dimensionality reduction, relying on the elicitation of implied patterns and insights. Nevertheless, issues also arise from the potential imbalance of the available data, sometimes—in contrast with other cases—aggravated by the lack of sufficient relevant features to safely infer the underlying connections between the characteristics and corresponding classification of examples, as well as from potential bias, inhomogeneity, and, in general, non-uniform data distribution. As such, the challenges posed by urban data require innovative methodologies capable of handling these unique characteristics while maintaining a comprehensive understanding of the urban landscape, thus calling for experimentation with alternative arrangements of machine learning methods in novel scenarios and combinations. The following chapters detail such an approach, aiming to tackle urban data challenges through the adoption of advanced machine learning mechanisms, including convolutional neural networks, as well as data visualization in a graph-based form to assist and enhance the performance of these mechanisms.

### 1.3. Research Contribution

The current research contributes to the area of decision support in urban planning, introducing a novel configuration comprising a deep learning mechanism applied to input urban data, visualized through adopting a graph-based representation. This approach aims to take advantage of the inherently increased capability of convolutional neural networks to successfully classify image-based input data. To achieve this, the available features participating in the decision support process are encompassed through their visualization in a graph form that is subsequently used as the dataset for the mechanism’s training and classification process. The results reveal promising performance in comparison to earlier work stemming from the same dataset based on alternative machine learning mechanisms that rely on the input data being in traditional tabular form.

## 2. Related Work

For a more comprehensive analysis of the challenges in the aforementioned scientific domains, it is imperative that this research is placed in context with other works, especially in the fields of urban planning, convolutional neural networks, and their coalescence.

### 2.1. Urban Planning Challenges

Silva et al. [8] note that the challenges caused by the heterogeneity of urban big data embrace the need for the use of sophisticated tools and methodologies aimed at integrating and making sense of vast amounts of diverse datasets emanating from urban areas that are highly heterogeneous. Convolutional neural networks (CNNs) represent such a candidate tool. Hamilton et al. [9], in their work on spatial data, accentuate the necessity for models capable of describing complex relations, introducing GNNs for that purpose. Chen et al. [10], in their work, “Machine Learning in Urban Planning”, engage the transformative potential of data-driven methodologies for the decision making domain, paving the way for the use of CNNs. Krizhevsky et al. [11], in their work perform image classification using convolutional neural networks (CNNs). Another important extension of neural networks’ capabilities for modelling the complex relationships within non-Euclidean domains has been created by Velickovic et al. [12] through their research on Graph Attention Networks, thus highlighting the importance of these methodologies to the spatial and relational intricacies of urban data. The aforementioned works demonstrate that CNNs can be efficient for processing visual data, rendering them as indispensable in geo-spatial-related tasks and urban imagery analysis

### 2.2. Convolutional Neural Networks’ Architecture

Convolutional neural networks (CNNs) have arisen in the context of image classification as a powerful deep learning mechanism able to adapt and conform to the intricacies of visual input data [13]. They achieve this based on an architecture that comprises an input stage and an output stage of neurons, interposed by multiple hidden layers, responsible for the feature isolation and exploitation of the input image. The latter tasks are distributed in a series of layer groups, responsible for feature amplification, isolation, and reduction, respectively. The first of these layers modifies the input image through a series of convolution steps, amplifying the most prominent features to render them significant for the next stage of feature isolation. This is achieved by focusing on smaller parts of the input image for feature learning and identification through localized filtering convolution operations. The second stage eliminates non-significant features and yields the results for the third stage of dimension reduction. This stage aims to reduce the features participating in the learning task, thus allowing for a simpler mechanism to be trained and subsequently used in the overall classification process while improving robustness against small perturbations of the input.

The aforementioned stages form a repetitive process, yielding a minimal set of features that adequately represent the original input image. In practice, numerous hidden layers are used, incorporating these three stages repeatedly in order to focus and isolate distinct features and compress these features into a limited set that still encompasses the original information to an adequate degree. Following this repetitive process, the minimal feature set is used for training and classification tasks. The final traditional fully connected neural network layer provides the probability for each alternative class in relation to the input image, thus offering the eventual classification as the output.

### 2.3. CNNs in Urban Planning

CNNs have been employed in the area of urban planning but typically in contexts where the original input dataset is already image-based. In particular, Huang et al. [14] perform urban land-use mapping based on multispectral remote sensing images using a convolution neural network appropriately modified to address the increased number of channels of the image input. Yang et al. [15] employ a convolutional neural network to predict restaurant locations, relying solely on 2D images revealing the outline of the buildings being classified. Chen et al. [16] utilize a channel-expanded CNN (CECNN) to tackle noise impeding the precision of urban green areas appearing in satellite images. Bao et al. [17] address the problem of recognition of urban functional zones, based on remote sensing data and semantic information, employing a deeper-feature convolutional neural network (DFCNN) to infer features and semantic information from the available images, enhancing boundary extraction. A common characteristic of the aforementioned approaches is the presence of images as raw datasets, thus directly suggesting the potential benefits of CNNs, whereas in the current work, the datasets comprise a multitude of topographic and semantic information that is transformed into graph representations, thus eventually rendered accessible to CNN classifiers.

## 3. Premise Outline and Principles

Our research work maintains the principle of operating on urban data from a multitude of sources that are all openly available to the public. The former fact ensured that we addressed the typical heterogeneity of data addressed in urban planning problems [5,6], while the latter ensured the potential, for any interested researchers, to replicate our results as well as expand them, invoking alternative techniques and configurations. The homogenization of these data rendered them accessible to the next stage, which was the choice of a typical urban planning problem as the canvas for the experimental evaluation of alternative classifiers. The chosen urban planning problem comprised the selection of an appropriate location/building for a parking facility, posing as a typical representative of this category of problems [3,4] and motivated from the semantic content per se of the aforementioned available datasets, which, for the specific problem, bore enough range to support the relevant classification endeavor, offering an adequate variety of features contributing to the decision task at hand.

Having previously focused on the most prominent classifiers and isolating random forests as the best performing representative [18], we have chosen to enhance the palette of classifier mechanisms to be employed by harnessing the inherent visual qualities of the participating urban data. In order to achieve this, we have transformed each representative of the participating locations into a graph, incorporating in the visual representation all relevant information, thus rendering the samples accessible to convolutional neural networks as the most prominent representative of visual information classifiers [11]. For this experimentation, we have employed three alternative families of CNN implementations [19,20,21] while also maintaining the principle of availability towards replication of results and further research.

## 4. Problem Data Collection and Preprocessing

### 4.1. Problem Formulation

As a continuation of our prior work [18], we maintained the use of our system and the same data sources, but enriched them with additional new information consisting of real-world open urban data to power this experiment. For the initial step, the data derived from the city of Lyon. The dataset comprised around 800,000 buildings, including water masses, parks, and forests, obtained from the open data service of the city [22]. The data were originally collected in 2019 for the needs of the previous work, but as they relate to buildings, parks, etc., it is reasonable to consider them still accurate and relevant. In addition, bicycle rental stations were added subsequently (2024).

Upon thorough examination of the data, we observed a considerable amount of geographic and semantic information related to parking spaces in the vicinity of the preponderant area we selected for our experiment. The suitability of a given site for use as a parking facility is an inquiry that fits the requirements of an urban planning problem, whilst also attracting significant commercial interest. The existence of a tool that may indicate a probable appropriate use of a space or building, or anticipate the suitability of it for a certain purpose, can be a valuable decision support tool for an expert, which was a part of the goal of our prior work. More specifically, we employed machine learning techniques with the random forests classifier as the dominant method, in a system that combines, blends, and merges various types of data from different sources, encodes them using a semantic model that can capture and utilize both low-level geometric information and higher-level semantic information, and subsequently feeds them to the classifiers. Several experiments were conducted with classifiers (indicatively, we mention KNN, SVM, Naïve Bayes, neural networks, random forests, and bag of decision trees). 

The acquisition of real-world parking data, even those enriched with spatial and semantic features, does not imply that we automatically understand the necessary information included therein in terms of the factors that contribute to making a parking lot useful, vital, or profitable. Essentially, this means that the feature extraction process is not a trivial process. In this context, a variety of factors will be investigated as potential descriptors, including distance from landmarks, distance from other parking spots, density of occurrence per specific area, distance from modes of public transportation and their plurality in a given area, and distance and density of occurrence in relation to touristic, economic, and monetary points of interest, among others.

### 4.2. Description of the Dataset and Data Collection

The initial step of the first stage involves collecting and processing the data in order to prepare the data for their import to our system. The system we used allows and provides for the input of different types of data originating from different sources and consequently diverse formats, such as raw datasets, provided as plain files or in a more difficult-to-exploit form, through application programming interfaces (APIs) bestowed by web platforms, or even online map services.

Wherever possible, to offset the risks arising from the use of open data that we mentioned above, the candidate datasets are put in comparison. Where there are multiple sources for a certain area, a cross-check of the information is performed and the components—the matching buildings—are forwarded to the next control stage and placed in the database. If a difference occurs, visual inspection is then performed, and, along with other criteria such as the date of acquisition, they are aggregated to arrive at the final conclusion.

Data entry does not guarantee validity since the data are georeferenced and may be encoded in different ways and in a different coordinate system depending on the organization providing them; therefore, several checks are performed that control and convert, if necessary, the data into a common coordinate system.

There is a very small percentage of data (in our test’s case, 4–6 buildings out of 800,000) that during the conversion presents errors and incorrect geometrical characteristics, and which is inevitably rejected. However, this percentage is negligible (0.00000625%), as it affects neither the size of the set nor the credibility of the method.

The next step is the normalization and homogenization of the coordinate system for each of the various data segments, so that geometric operations and geographic correlations can then be applied to correctly update the system and subsequently feed and inform the ontological model with the correct information. Each organization provides its data in different formats and diverse systems of geographical coordinates. The data are subjected to specific queries and appropriately converted to abide by the rules of the geodatabase and the semantic model used. Finally, appropriate queries are used to extract semantic information from the data.

**Figure 1:**
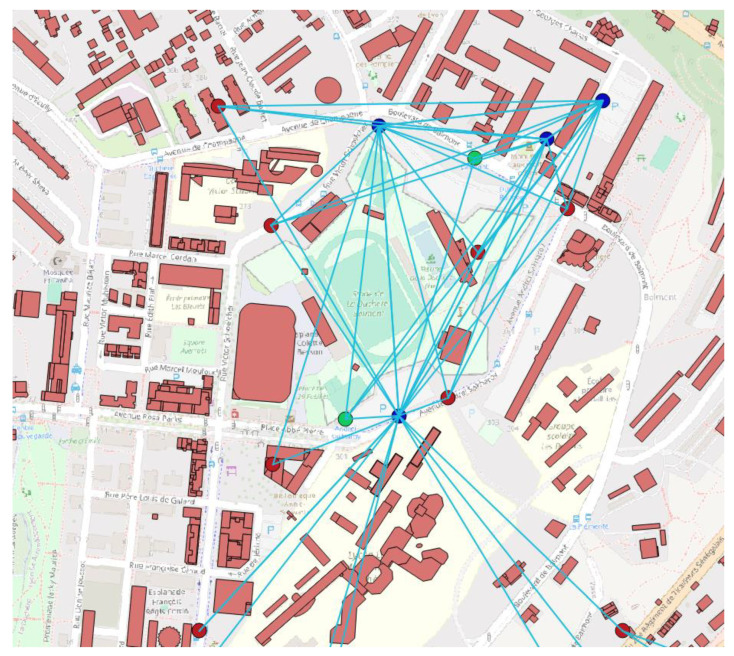
Aspect of the visual representation of the related points of interest: blue circles indicate parking lots, red circles indicate points of touristic interest and green circles indicate bike rental points. The lines connecting them indicate that the Euclidean distance between them is less than 500 m. Street names and locations of Lyon, contained in the map excerpt, appear in French.

After the aforementioned processing, cleaning, and distillation of the data, the semantic information on the use of buildings as parking facilities emerges. As mentioned in a previous paragraph, we are unable to know or be certain of the factors that make a location suitable for use as a parking facility, but it is reasonable that it should be related to the presence, distance metrics (both Euclidean and Dijkstra—shortest travelling distance using the road network), and quantity of other parking facilities in the area, and the same applies to points of touristic interest, bank ATMs, and bicycle rental stations. Finally, the building area is also an important factor and acts as a quantitative indicator. An example of the visualization of the process of this stage can be seen in Figure 1.

### 4.3. Graph Preprocessing

After extracting the required data and metrics from our system, they were used as input data for the next module. In our previous work, we had studied and used several classifiers: indicatively worth mentioning are random forests, Support Vector Machines (SVM), feedforward neural networks (multilayer perceptrons), bag of decision trees, k-Nearest Neighbors, and Naïve Bayes. In the present paper, we explore the potential benefits arising from representing the aforementioned data in the form of graphs before subsequently presenting them into a convolutional neural network.

The first step resides in the translation of each building into an individual graph structure, with edges forged in accordance with and in proportion to the spatial relationships embedded in the dataset features. Other researchers have also brought into attention the efficacy of graph-based representations in capturing complex dependencies and patterns within spatial data. The work of Kipf and Welling [23] emphasizes the utility of Graph Neural Networks (GNNs) in modeling relationships in non-Euclidean domains, precisely the particular complexities associated with urban spatial datasets. The construction of graphs, with buildings as nodes and edges denoting spatial distances, aligns with the principles of graph theory, providing a more comprehensive representation of the urban environment.

**Figure 2:**
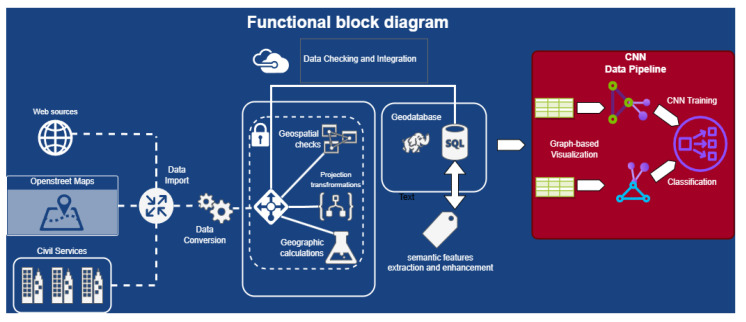
Data pipeline process block diagram.

The rationale for adopting graph-based representations is further underscored by the nature of urban spatial dependencies. Traditional machine learning models, designed for tabular data, may struggle to encapsulate the intricate relationships among buildings and their functional roles within an urban landscape. Graph structures inherently encapsulate spatial dependencies, allowing the model to discern nuanced patterns that extend beyond numerical proximity. This approach draws inspiration from the success of graph-based methods in capturing relational dependencies in diverse domains, as evidenced by the comprehensive survey by Battiston et al. [24] on the role of graph theory in understanding complex systems. The whole process can be summarized in the following block diagram (Figure 2).

Moreover, the incorporation of distance in the edges is consistent with the fundamental principles of urban planning, where proximity and connectivity have critical importance in spatial relationships. The Euclidean and Dijkstra calculated distances encapsulate both geometric and real-world travel considerations, adding a layer of realism to the graph representation. This hybrid approach, combining geometric and functional aspects, resonates with the nature of urban spatial relationships, as mentioned by Buttenfield and McMaster [25] in their work on spatial models in geography. Therefore, the conversion of features into graph structures is not just a technical task but a contextually appropriate transformation that enriches the dataset for subsequent analysis with a CNN.

## 5. Graph-Based Visual Representation

This chapter will address the process of graph representation of the aforementioned urban data. More specifically, the parameters we want to encode, as mentioned in the previous chapters, are the number of buildings of the same type (parking or false parking) within a given distance (500 m), as well as the distance from each such building located within this range, while similarly repeating this for ATMs, points of touristic interest, and bicycle rental stations.

**Figure 3:**
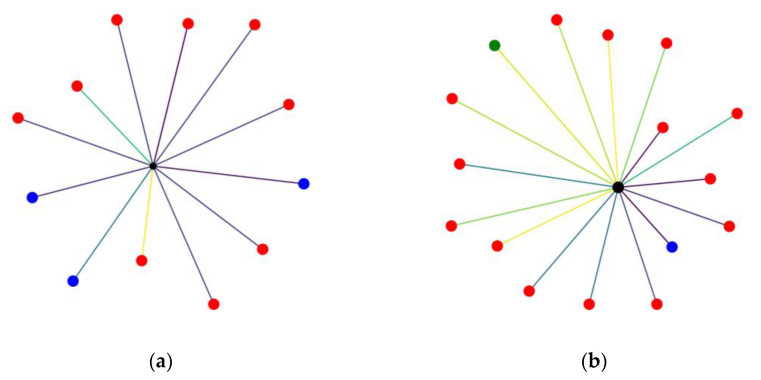
Representative visualization samples from each category, where the difference in distance, plurality of representative neighboring features, and area are evident. (**a**) Graph-based visualization of a parking building (positive classification sample); (**b**) graph-based visualization of a false parking building (negative classification sample). In both samples the central black circle is representative of the site under classification, while the other circles follow the same color scheme adopted in Figure 1. The color of the edges is determined by their weight according to a standard colormap.

The foundation of our graph-based representation is derived from the intricate relationships among urban features. In an effort to capture and integrate all of the aforementioned features, a graph and its visualization is created for each representative building of interest (parking or false parking) according to the following procedure, as seen in Figure 3: In the center of the graph resides the building for which the graph is constructed, and nodes are added for each of the other characteristic reference points mentioned (other parking buildings, ATMs, points of touristic interest, etc.). The nodes are colored in accordance with their type. Furthermore, depending on their distance from the reference point, a color mapping representation is maintained, thus ensuring that this spatial information is preserved in the transformation. The edges connecting nodes with greater distance from the reference point will appear in a different color to the ones with lesser distance. Additionally, in order to incorporate the metric of area in the visual representation of the graph, the size of the central node that depicts the point of interest is visualized in proportion to its area, setting some upper and lower thresholds to ensure visibility on all occasions without significant alteration of the result.

This new transformed dataset is exploited for the continuation of the experiment. For the classification, deep learning techniques will be used: more specifically, convolutional neural networks. Deep learning is a subdomain of machine learning. The former utilizes neural networks with a considerable number of layers to perform the modeling of data.

## 6. Experimental Setup

**Figure 4:**
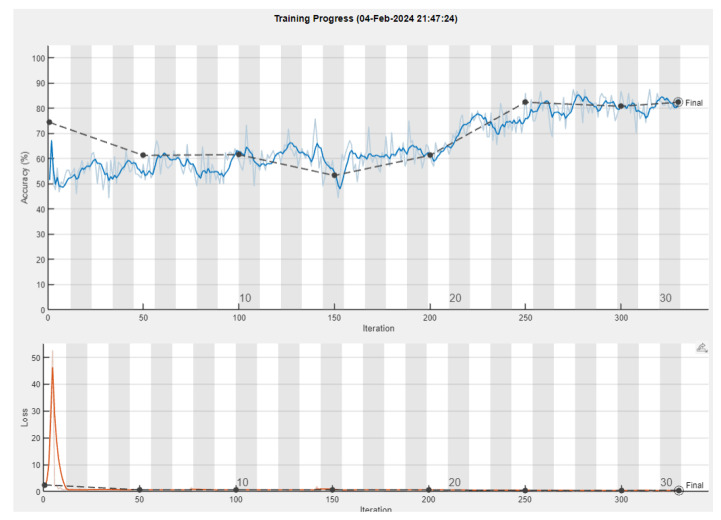
Training plot for AlexNet. The upper diagram illustrates the accuracy as it is formed after each training epoch, where the blue line represents the training accuracy, and the dark line represents the validation accuracy. The lower diagram depicts the loss, where the orange line represents the training loss and the dark dotted line represents the validation loss.

As mentioned in previous sections, upon constructing the graphs from urban features, the ensuing experimental setup entails the transformation of these graphs into images and then delineates two distinct approaches. These images, serving as inputs, are then subjected to several convolutional neural networks (CNNs) (Figure 4) for training and testing.

### 6.1. Experiment CNN Architectural Overview and Implementation

Convolutional neural networks are a subclass of deep neural networks, which in turn are a subset of neural networks, with their main distinguishing feature being their depth. The latter have numerous layers residing in their architecture, including fully connected, convolutional, pooling, and recurrent layers, which enable the network to learn complex patterns and representations from the input data. Their distinguishing feature that endows them the denomination “deep” is signified by the presence of multiple hidden layers between the input and output ones. Each of these hidden layers transforms data into a more abstract and composite representation. Their network identifies the features and patterns required to generate predictions or make decisions as the data travel through these layers, constituting them particularly powerful for tasks like image recognition.

Convolutional neural networks (CNNs) are a subtype of Deep Neural Networks that excel at processing grid-like topology data, such as images. The pivotal distinction between CNNs and other Deep Neural Networks is their reliance on convolutional layers. Correspondingly, these layers employ a mathematical operation called convolution, in which a filter or kernel “slides” over the input data (e.g., an image) to create feature maps and thus capture spatial dependencies in an image by applying appropriate filters.

In contrast to fully connected layers, where each neuron is connected to every neuron in the previous and next layers, convolutional layers only have a small region of the input connected to each neuron. This local connectivity and the shared weights result in translation invariance, making them more efficient and reducing the number of parameters needing to be learned.

In selecting specific convolutional neural networks (CNNs) for our experiment, it is important to consider criteria beyond architectural complexities. Decisions stem from an amalgamation of architectural features, computational efficiency, and task-specific requirements. AlexNet, revered for its pioneering contributions to the field, remains a stalwart choice due to its hierarchical structure and robust performance in image classification tasks [20]. Its architecture, characterized by convolutional and max-pooling layers followed by densely connected layers, enables effective feature extraction and hierarchical representation learning. Furthermore, the utilization of rectified linear units (ReLUs) as activation functions and dropout regularization techniques enhances the model’s robustness and mitigates overfitting, rendering AlexNet a strong candidate.

SqueezeNet offers an equally compelling alternative that performs well in computationally constrained scenarios [21]. It can avoid the computational overhead typically associated with larger models by incorporating only a fraction of the parameters and size of traditional CNN architectures. Using fire modules that incorporate both squeeze and expand layers, the architecture is designed to be efficient and provides real-time inference without sacrificing performance, thus rendering this approach to model design appealing.

VGG-16, on the other hand, epitomizes a middle ground between the architectural profundity of AlexNet and the computational efficiency of SqueezeNet. VGG-16 features a homogeneous architecture comprising stacked convolutional layers with small 3 × 3 filters, interspersed with max-pooling layers for spatial downsampling [19]. This uniformity and depth contribute to facilitating the learning of rich hierarchical representations, which in turn leads to enhanced generalization performance across an extended range of datasets. Despite its computational demands, VGG-16 remains a cornerstone in the CNN field, offering a versatile platform for deep feature learning.

### 6.2. CNN Evaluation

The first approach is to present the entirety of the images to the networks, use the integrated functions of the tools to automatically separate the data into training and validation sets, and then calculate their efficiency. The second approach for further scrutinization of the networks involves completely masking a larger portion of the data so that the training is performed with fewer samples, and then measure its performance.

For this, we let the set of buildings be

$$B=\left\{b_{1},b_{2},\ldots ,b_{k},k=total\;number\;of\;buildings\;in\;database\right\}.$$

In order to be able to apply and evaluate the intended machine learning configuration, representatives of the two classes are required to be subjected to the aforementioned procedure. In the current experiment, we focus on the suitability of a building to qualify as a parking facility. With respect to the need for positive examples and negative examples, we have used the real-world information extracted by our system concerning the actual use of buildings marked as parking lots as the positive class. Therefore, for the positive examples we have

(1) $$P=\left\{p\in B,u\left(p\right)=parking\right\},$$

where *u(p)* indicates the use of the building of interest. Likewise,

(2) $$N=\left\{n\in B,u\left(n\right)\neq parking\right\}.$$

This holds that B=P∪N.

Potentially, any of the buildings in *N* could be used as negative examples. However, to avoid problems arising from imbalanced datasets, we chose to use the majority of the members of *P* as positive class representatives in our experiments, while creating a dataset of randomly selected non-parking buildings as negative class representatives. Positive examples correspond to actual parking areas distributed across the region of interest. The number of recorded real parking buildings in our dataset is 1000.

In the following presentation of the assessment of the results, we have adopted the following terms:

- True Positives (*TPs*): $TP=P_{c}\cap P_{e}$
- True Negatives (*TNs*): $TN=N_{c}\cap N_{e}^{i}$
- False Positives (*FPs*): $FP=P_{c}\cap N_{e}^{i}$
- False Negatives (*FNs*): $FN=N_{c}\cap P_{e}$

The above are summarized in the following table (Table 1):

For the evaluation of the results, the following metrics are used:

- Accuracy:

(3) $$Accuracy=\frac{Number\;of\;correct\;predictions}{Total\;number\;of\;predictions\;made\;}=\frac{\left|TP\right|+\left|TN\right|}{\left|P_{c}\right|+\left|N_{c}\right|}$$

- Specificity:

(4) $$Specificity=\frac{False\;Positive}{False\;Positive+True\;Negative}=\frac{\left|FP\right|}{\left|N_{e}^{i}\right|}$$

- Precision:

(5) $$Precision=\frac{True\;Positive}{\;True\;Positive+False\;Positive}=\frac{\left|TP\right|}{\left|P_{c}\right|}$$

- Recall (or Sensitivity):

(6) $$Recall=\frac{True\;Positive}{\;True\;Positive+False\;Negative}=\frac{\left|TP\right|}{\left|P_{e}\right|}$$

- F1 measure: The F1 measure is the harmonic mean between Precision and Recall. Its range is [0, 1]. It provides information on how precise the classifier is (how many instances it classifies correctly), as well as how robust it is (if it misses a significant number of instances).

(7) $$F1=2\frac{1}{\;\frac{1}{Precision}+\frac{1}{Recall}}$$

- G-mean: The geometric mean (G-mean) is the root of the product of the class-wise Sensitivity. This measure tries to maximize the Accuracy for each of the classes while keeping the Accuracy values balanced. For binary classification, G-mean is the squared root of the product of the Sensitivity and Specificity.

(8) $$G\;Mean=\sqrt{Sensitivity\cdot Specificity}$$

### 6.3. AlexNet CNN

AlexNet is one of the CNNs considered to be a pioneer in the image classification field, and its architecture laid the foundation for subsequent models. The latter comprises eight layers of learnable parameters, including five convolutional layers followed by max-pooling layers and three fully connected layers. The convolutional layers apply learnable filters to extract features from the input images, while the max-pooling layers reduce the spatial dimensions of the feature maps to improve computational efficiency and increase the receptive field of the network. The fully connected layers at the end of the network perform high-level feature aggregation and classification.

One of the key features of AlexNet is its use of rectified linear units (ReLUs) as activation functions, which help to alleviate the vanishing gradient problem and accelerate convergence during training. Additionally, the use of dropout regularization in the fully connected layers helps prevent overfitting by randomly dropping out units during training. Another distinct characteristic is the utilization of data augmentation techniques, such as random cropping and horizontal flipping, to increase the diversity of the training data and improve the generalization performance of the model.

For our experiment, the standard procedure was followed for all of the CNNs tested: namely, during training, optimization algorithms such as stochastic gradient descent (SGD) and Adam were used to try and minimize the loss function, and adjust the network’s weights and biases to minimize prediction errors (Figure 4).

**Figure 5:**
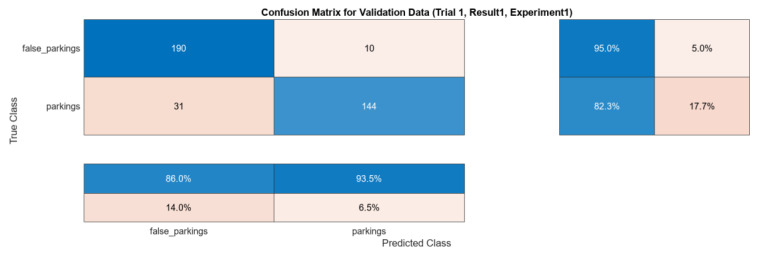
Confusion matrix for AlexNet. The colors in all matrices represent the values of the predictions. Darker colors (blue) indicate higher percentages, while lighter colors (pink) represent lower percentages. The main (**top-left**) matrix contains numerical information, whereas the sub-matrices (**bottom-left**, **top-right**) contain percentages, also rearranged to present successful classifications percentages closer to the main matrix.

The model’s performance was monitored during training using the validation set, and hyperparameters such as the learning rate and dropout rate were tuned to prevent overfitting. After completing the training, the model’s generalization performance was evaluated using the test set. The confusion matrix of the results can be seen in Figure 5.

### 6.4. SqueezeNet CNN

**Figure 6:**
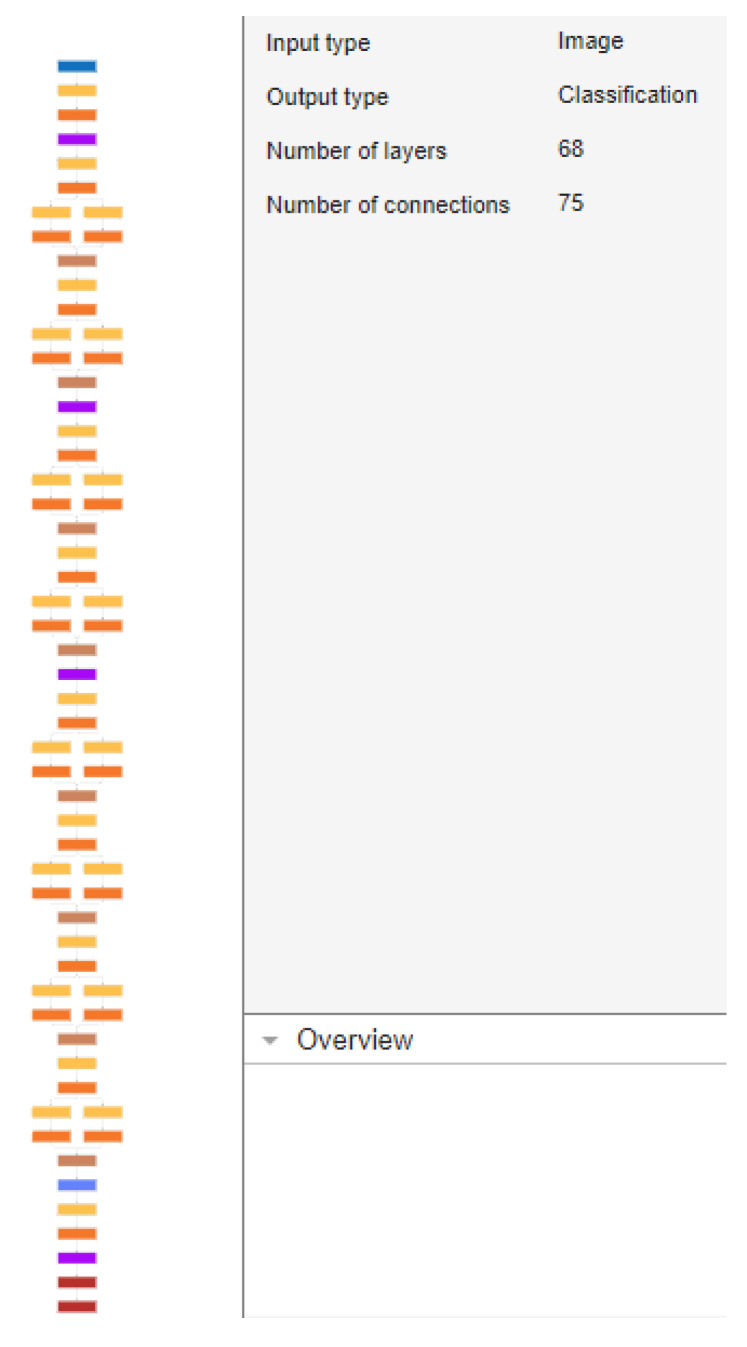
Architecture (layers and connections) of the SqueezeNet CNN.

SqueezeNet is a commonly used CNN aimed towards the classification of images, comprising a relatively small number of layers and connections yet maintaining a comparable accuracy. Some of its features include, except from the reduced parameter count, the “fire modules”, which contain a squeeze convolutional layer (using 1 × 1 filters) followed by an expand layer containing a combination of multiple 1 × 1 and 3 × 3 filters. The former is used to reduce the number of input channels or data depth, while the expand layer maps these compressed data into an increased-dimensionality space. This design allows for a reduction in parameters while still maintaining the capacity to extract complex features. It also includes a decreased usage of 3 × 3 filters to save more parameters through a wider use of 1 × 1 filters. Finally, apart from its novel architecture, SqueezeNet is highly susceptible to compression methods like deep compression, which further reduce its size with minimal loss of accuracy. A depiction of the architecture appears in Figure 6. Each layer is represented by colored blocks therein as follows:

- Blue: These blocks represent the input and output layers where the initial image data is fed into the network and the final classification is outputted.
- Orange: These represent ReLU (Rectified Linear Unit Layers.
- Purple: These indicate max-pooling layers used for down-sampling and reducing the spatial dimensions. They help to make the model more robust to variations in the input and reduce overfitting.
- Yellow: These depict connected layers that perform classification based on the features extracted by the previous layers.
- Dark Red: These are softmax used as the last activation function to normalize the output of a network to a probability distribution over predicted output classes.
- Brown: these represent DepthConcatenationLayer: In some network architectures, the outputs of several layers might be concatenated together along the channel dimension.

**Figure 7:**
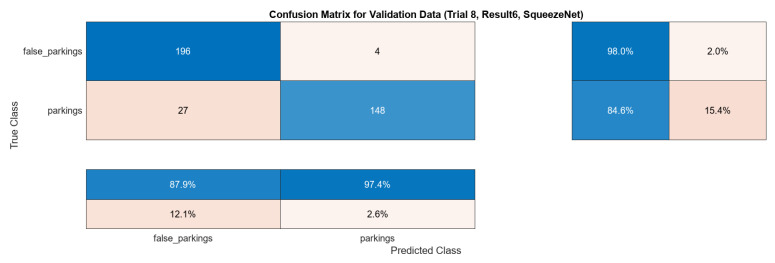
Confusion matrix for SqueezeNet. The colors in all matrices represent the values of the predictions. Darker colors (blue) indicate higher percentages, while lighter colors (orange) represent lower percentages. The main (**top-left**) matrix contains numerical information, whereas the sub-matrices (**bottom-left**, **top-right**) contain percentages, also rearranged to present successful classifications percentages closer to the main matrix.

**Figure 8:**
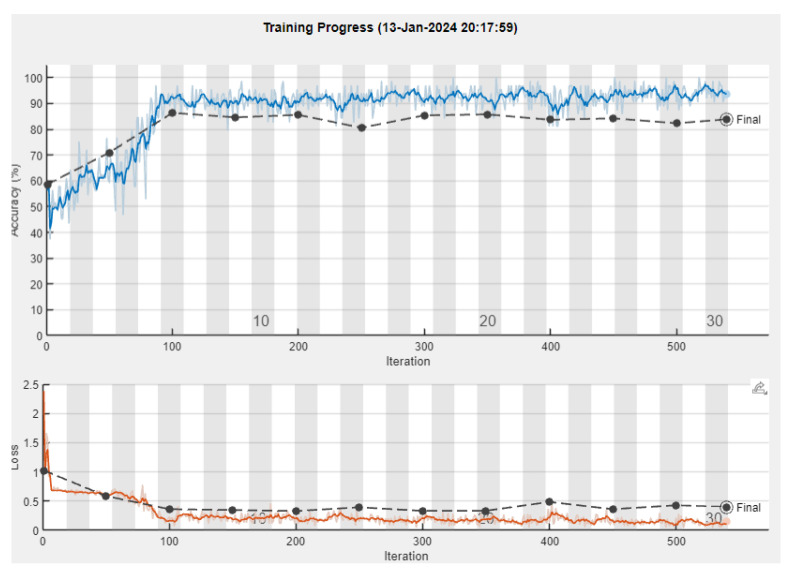
Training plot for SqueezeNet. The upper diagram illustrates the accuracy as it is formed after each training epoch, where the blue line represents the training accuracy, and the dark line represents the validation accuracy. The lower diagram depicts the loss, where the orange line represents the training loss and the dark dotted line represents the validation loss.

After performing the experiment, we acquired the following results depicted in Figure 7 and, in a more detailed form containing all of the metrics, shown in Table 2. The progress of the training can be observed in Figure 8.

It is worth noticing and commenting on the particularly high values of Recall, presenting the model’s ability to correctly identify the positive cases.

### 6.5. VGG-16 CNN

**Figure 9:**
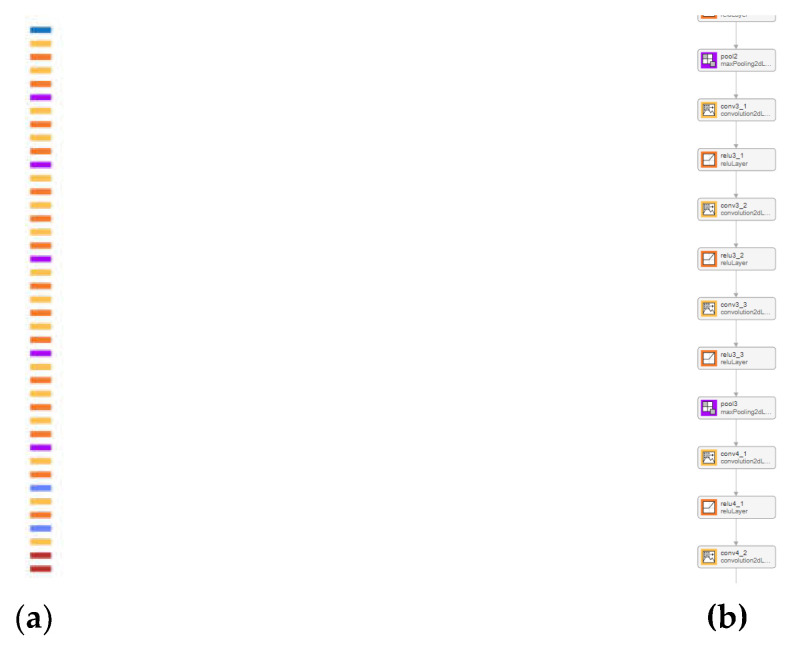
Depiction of the layers and architecture of the VGG-16 CNN used for classification. (**a**) Architecture of the CNN containing all of the layers; (**b**) magnified fragment demonstrating a partial view of the network layers and connections. The color scheme used is identical to the one detailed for SqueezeNet in Figure 6.

VGG-16 is one of the first convolutional neural networks that proved that adding more depth and layers to the network could improve performance. We can see a depiction of its architecture in Figure 9.

**Figure 10:**
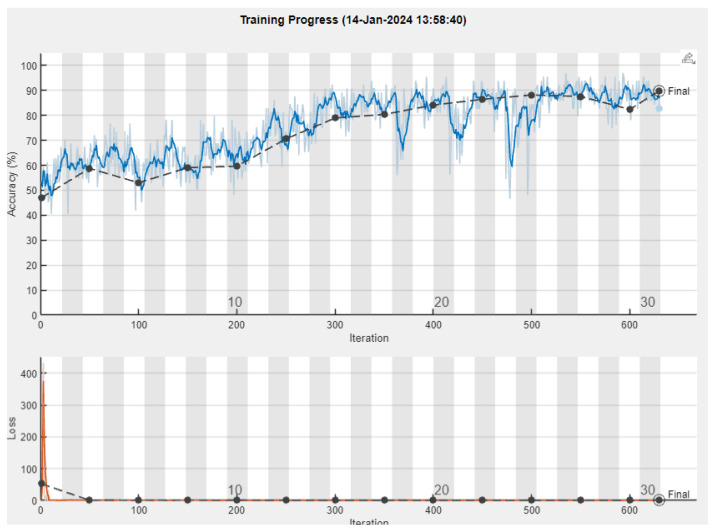
Training plot for VGG-16. The upper diagram illustrates the accuracy as it is formed after each training epoch, where the blue line represents the training accuracy, and the dark line represents the validation accuracy. The lower diagram depicts the loss, where the orange line represents the training loss and the dark dotted line represents the validation loss.

Upon conducting the experiment, we observed the results of the training process (Figure 10).

**Figure 11:**
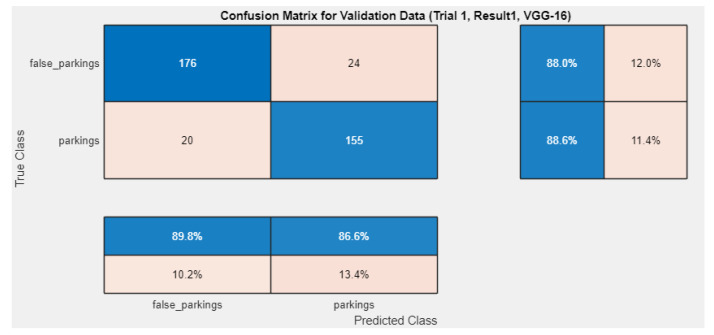
Confusion matrix for VGG-16. The colors in all matrices represent the values of the predictions. Darker colors (blue) indicate higher percentages, while lighter colors (orange) represent lower percentages. The main (**top-left**) matrix contains numerical information, whereas the sub-matrices (**bottom-left**, **top-right**) contain percentages, also rearranged to present successful classifications percentages closer to the main matrix.

Continuing the experiment, after carrying out the initial tests, we tried to optimize several hyperparameters like the solver, the mini-batch size, and the learning rate. Performing an exhaustive sweep and checking the performance, we observe that the use of sgdm as a solver produced far inferior results, while the mini-batch size produced better results, with a value of 64. In Figure 11, we can see the confusion matrix, while in Table 3, we can see all of the metrics.

### 6.6. Comparison with Results from Machine Learning Classifiers

In the following table (Table 4), a comparative chart of all of the metrics from the experiments is presented. The first entries concern classical classifiers and the conventional method, where the results are taken from our previous work [18], while the last three entries concern the current experiment with the CNN graph-based approach. We note that this is a promising method that demonstrates considerable results. In this process, numerous implementations of CNNs were tested, including custom ones, but since they produced inferior results, only the CNNs worth mentioning were included. It is in our plans to experiment with other CNN architectures and configurations in the future.

### 6.7. Discussion

Heterogeneous urban data, originating from various sources, pose a number of challenges before rendering themselves susceptive to machine learning classifiers. In the current work, we build upon the data preprocessing and homogenization methods we employed to evaluate a group of classifiers in our previous work [18] in order to explore a novel combination of CNNs and graph representation of the samples, operating on the same dataset. Random forests emerged as the most promising representative in our previous approach and thus serve as the baseline performance with which to compare the results of the current work.

Out of the six performance indices presented in Table 4, random forests outperform all CNN variations in three areas, namely Specificity, Precision, and the F1 measure, with a very small relative advantage, i.e., less than 1%, in one of the three. Examining each CNN variation individually, we observe that VGG-16, while better than all other classifiers evaluated in our previous work, underperforms when compared to random forests, regarding all indices. It is also the case that VGG-16 does not achieve in any index a small relative difference to the random forest equivalent, practically rendering it as the least promising of the examined alternatives in the current work. AlexNet clearly outperforms random forests in terms of Recall, also showing no relative difference anywhere else, while outperforming all other classifiers from our previous work.

The most prominent representative of the current approach appears to be the SqueezeNet CNN variation, which outperforms random forests in three indices, namely Accuracy, Recall, and the G-mean, having a similar marginal advantage in Accuracy as the random forests presented in the F1 measure. Moreover, we may observe that SqueezeNet presents the highest performance, in terms of absolute numbers, for the Recall index result.

Pondering on the collective performance levels of the CNN representatives against the random forests, it appears that a trend emerges favoring the latter in Specificity and Precision, while the former appear mostly superior in terms of Recall (excluding VGG-16). This observation leads to the consideration of their potential specialized usage for custom tasks focused on maximizing particular indices, employing the appropriate mechanism. Alternatively, their combined usage in certain premises would allow us to reap the benefits from both approaches and ensure increased overall performance.

## 7. Conclusions and Future Work

### 7.1. Conclusions

The current work exists in the context of urban planning decision support through the use of machine learning mechanisms operating on real urban data. Our approach has attempted to make already-available urban data accessible to a prominent representative of deep learning mechanisms and subsequently evaluate the latter’s performance in comparison with previously employed alternative machine learning approaches, applied on the same dataset, for decision support in a typical urban planning problem.

Our input data represented features of existing and candidate parking buildings, vectorized upon the corresponding information available for them in open-data repositories maintained by public organizations and official stakeholders (e.g., the Estate Property Agency). In order to streamline this configuration, the input data were transformed into a graph-based visualization, carrying the entire semantic content of the original dataset in a visual form. Based on the transformed dataset, a convolutional neural network (CNN) was trained and subsequently invoked to suggest the classification of graph-represented visual samples modeling positive and negative examples of parking buildings. The experimental results are promising in comparison to previous efforts on the same dataset and reveal the benefits and favorable potential of urban data graph-based visualization that renders these data accessible to relevant deep learning mechanisms.

### 7.2. Future Work

The graph-based visualization of the input dataset has allowed successful invocation of relevant deep learning algorithms, thus setting the path for further investigation of the proposed and similar alternative configurations. Alternative learning mechanisms and visual representations are to be adopted anticipating enhancement of the achieved results. Such mechanisms include, without being limited to, Graph Neural Networks (GNNs) and their variations, offering further exploration of the graph representation and the positive potential of its application to decision support processes that have to rely on the relevant urban data. Furthermore, the continuation of this experiment with different and diverse datasets could provide useful insights on the range of applicability and robustness of the invoked mechanisms against input diversity and larger perturbations.

In parallel, trained versions of these learning mechanisms are to be integrated to the existing decision support environment [18], enhancing urban planning functionality by providing the relevant classification services. The potential of offering expert users alternative visualizations of their input data in connection with the graph-based representation and the experimental setup presented herein will be explored in future work.

## Figures and Tables

**Table 1:** Summary of prediction results.

		Actual Class	
		$\mathrm{YES}\;(P_{e}$)	$\mathrm{NO}\;(N_{e}^{i}$)
**Classifier’s Prediction**	$\mathrm{YES}\;(P_{c})$	TP	FP
	$\mathrm{NO}\;(N_{c})$	FN	TN

**Table 2:** Results for the SqueezeNet CNN.

Accuracy	Specificity	Precision	Recall	F1 Measure	G-Mean
0.917	0.88	0.85	0.97	0.91	0.93
0.912	0.88	0.85	0.96	0.90	0.92

**Table 3:** Results from the VGG-16 CNN.

Accuracy	Specificity	Precision	Recall	F1 Measure	G-Mean
0.882	0.90	0.89	0.87	0.88	0.88

**Table 4:** Comparison of metrics for all classifiers.

Classifier	Accuracy	Specificity	Precision	Recall	F1 Measure	G-Mean
MLP	0.681	0.755	0.719	0.608	0.655	0.674
SVM	0.799	0.865	0.846	0.733	0.784	0.796
KNN	0.699	0.914	0.850	0.485	0.617	0.665
Naive Bayes	0.736	0.798	0.770	0.674	0.718	0.733
Bag of Decision trees	0.651	0.601	0.636	0.700	0.666	0.649
Random Forest	0.913	0.938	0.934	0.889	0.910	0.912
AlexNet	0.891	0.860	0.823	0.935	0.875	0.897
SqueezeNet	0.917	0.878	0.846	0.974	0.905	0.925
VGG-16	0.882	0.898	0.886	0.866	0.876	0.882

## Data Availability

Data supporting the findings of this study are publicly available through https://data.grandlyon.com/portail/en/recherche (accessed on 20 January 2024) and upon request from the corresponding author.
